# Seroprevalence of *Chlamydia abortus* infection in yak (*Bos grunniens*) in Tibet, China

**DOI:** 10.1186/s13620-021-00199-x

**Published:** 2021-06-30

**Authors:** Lin Liang, Yuan Wen, Zhaocai Li, Ping Liu, Xing Liu, Shuming Tan, Donghui Liu, Jizhang Zhou, Dewen Tong

**Affiliations:** 1 College of Veterinary Medicine, Northwest A&F University, 712100 Yangling, China; 2 State Key Laboratory of Veterinary Etiological Biology, Lanzhou Veterinary Research Institute, Chinese Academy of Agricultural Sciences, 730046 Lanzhou, China

**Keywords:** *Chlamydia abortus*, Seroprevalence, Yak, Tibet

## Abstract

*Chlamydia* spp. are prevalent zoonotic pathogens that infect a wide variety of host species. *Chlamydia abortus* (*C. abortus*) infection in yaks has been reported in Gansu and Qinghai province, China. However, no data about *C. abortus* infection are available in yaks in Tibet, China. A total of 938 serum samples was collected from yaks in Tibet, China and specific antibodies against *C. abortus* were detected by the enzyme-linked immunosorbent assay (ELISA). The results showed that the overall seroprevalence of *C. abortus* in yaks was 104/938 (11.1 %, 95 % confidence interval [CI] 9.1–13.1). The prevalence in female and male yaks was 59/556 (10.6 %, 95 % CI 8.0-13.2) and 45/382 (11.8 %, 95 % CI 8.5–15.0), respectively with no significant difference (*p* > 0.05). The seroprevalence of antibodies to *C. abortus* in yaks ranged from 8.0 to 18.2 % among the six different areas, and the difference was also without statistical significance (*p* > 0.05). The prevalence among different age groups ranged from 7.0 to 15.9 %, with a higher prevalence among 1 to 2 years age category. The results demonstrate the presence of *C. abortus* infection in yaks in Tibet and may pose a risk for the general yak populations in addition to its potential impact on public health and the local Tibetan economy. To our knowledge, this is the first seroprevalence survey of *C. abortus* in yaks in Tibet, China.

## Introduction

*Chlamydia* spp. are obligate intracellular bacteria with a unique biphasic developmental cycle, which can infect a wide range of animals and humans [1]. Eleven species of the family *Chlamydiaceae* have been identified along with six newly characterized candidate species [2–6]. Among them, infections in cattle with *Chlamydia* (*C*). *abortus*, *C. pecorum*, *C. psittaci*, *C. suis*, *C. gallinacea and C. pneumoniae* have been reported in many countries around the world, such as Australia, Ireland, Zimbabwe, Poland and China [7–12]. *C. abortus* is an important pathogen in cattle, capable of causing, premature birth, stillborn or weak offspring [8, 13]. It can also cause zoonotic infection, especially in pregnant women, where infection can lead to pelvic inflammatory disease, placental dysfunction and late-term fetal death [14].

There are approximately 14 million yaks in the Qinghai–Tibetan Plateau area of China, representing about 90 % of the yak population in the world. The yak is very important to native Tibetans, because of its high-quality fur, wool, meat and milk. *C. abortus* is an important pathogen of yak, capable of causing abortion in pregnant yak [15]. While data on the seroprevalence of *C. abortus* in yaks is available for Qinghai and Gansu provinces of China [16, 17], there is no information on the prevalence of *C. abortus* is in yaks in Tibet, China to the best of our knowledge. In this study, we investigated the seroprevalence of *C. abortus* and risk factors including location, gender and age of yaks in Tibet.

## Materials and methods

The study was carried out in Nierong county and Seni district of Naqu city, north Tibet in June 2019. Naqu City has an average altitude of 4,500 m. It is the highest place in China and called “the roof of the world”.

A total of 938 blood samples were collected randomly from apparently healthy yaks. Four hundred were collected in Nierong County area (Xiaqu, Seqing), including 247 females and 153 males while 538 samples were collected in the Seni district (Luoma, Namaqie, Dasha, Mufa), including 309 females and 229 males. The blood samples were placed at room temperature for 4 h and quickly transferred to the laboratory, and centrifuged at 3000 g for 10 min to obtain sera that were stored at -80 ℃ until use.

Anti-*C. abortus* IgG antibodies were detected with ELISA kits according to the manufacturer’s instructions (ID Screen® *Chlamydophila abortus* Indirect Multi-species, IDVET Innovative Diagnostics, Montpellier, France). The optical density (OD) was measured at 450 nm by an ELISA microplate reader (Multiskan™ FC, Thermo Fisher Scientific™, USA). Signal to noise ratio was calculated according to the following formula:

S/P (%) = 100 × (OD sample / OD positive control).

Where S: tested sample, P: positive control.

Sera samples with S/P ratios higher than 60 % were considered positive.

The differences in infection rate based on location, genders and age groups of yaks were analyzed by Chi-square test in SPSS (Statistical Analysis System, Version 22.0). Values of *p* < 0.05 were considered statistically significant.

## Results and discussion

Of the 938 serum samples, 104/938 (11.1 %, 95 % CI 9.1–13.1) were positive for *C. abortus* including 59/556 (10.6 %, 95 % CI 8.0-13.2) female yaks and 45/382 (11.8 %, 95 % CI 8.5–15.0) male yaks (Table 1), the difference, however, was not statistically significant (*p* > 0.05). Antibodies against *C. abortus* in yak varied from 8.0 to 18.2 % among the different regions but the difference was not statistically significant (*p* > 0.05) (Table 2). The seroprevalence among the different age groups ranged from 7.0 to 15.9 % (Table 3) and was higher in the 1 to 2 years category, but was not significantly different (*p* > 0.05).

**Table 1:** Prevalence of *Chlamydia abortus* infection in yaks by gender in Tibet, China

Gender	Number Tested	Number Positive	Prevalence % (95 % CI)	*p*
Female	556	59	10.6 (8.0-13.2)	0.575
Male	382	45	11.8 (8.5–15.0)	
Total	938	104	11.1 (9.1–13.1)

**Table 2:** Prevalence of *Chlamydia abortus* infection in yaks by areas in Tibet, China

	Area	Number Tested	Number Positive	Prevalence % (95 % CI)	*p*
Seni	Luoma	116	10	8.6 (3.4–13.8)	0.078
	Namaqie	161	19	11.8 (6.8–16.8)	
	Dasha	161	22	13.7 (8.3–19.0)	
	Mufa	100	11	11.0 (4.8–17.2)	
Nierong	Xiaqu	99	18	18.2 (10.5–25.9)	
	Seqing	301	24	8.0 (4.9–11.1)	
	Total	938	104	11.1 (9.1–13.1)

**Table 3:** Prevalence of *Chlamydia abortus* infection in yaks by age in Tibet, China

	Age group	Number Tested	Number Positive	Prevalence % (95 % CI)	*p*
	1 < age ≤ 2	107	17	15.9 (8.8–22.9)	0.412
	2 < age ≤ 3	129	18	14.0 (7.9–20.0)	
	3 < age ≤ 4	174	18	10.3 (5.8–14.9)	
	4 < age ≤ 5	143	17	11.9 (6.5–17.3)	
	5 < age ≤ 6 year	181	16	8.8 (4.7–13.0)	
	6 < age ≤ 7	133	13	9.8 (4.7–14.9)	
	age > 7	71	5	7.0 (0.9–13.1)	
	Total	938	104	11.1 (9.1–13.1)	

According to our results, the seroprevalence of yak *C. abortus* in Tibet was lower than that in Qinghai (17.66 %) and Gansu provinces (16.22 %) of China [16, 17], and in India (35 %) [18], but higher than the prevalence reported in Ireland and Poland [8, 11]. This difference may be related to the sample collection, feeding method, species and age of animals, as well as different detection methods, such as indirect hemagglutination assay (IHA) and ELISA which can cause a significant difference in seroprevalence rates because of differences in test sensitivity.

There was no significant difference in the prevalence in female and male yaks (*p* > 0.05), which was consistent with the results of white yaks in Gansu [17] and yaks in Qinghai Province [16]. The same results have been observed in studies on other animal species such as the Tibetan pigs in Tibet, China [19]. These findings suggested that gender might not be a key factor in *C. abortus* infection in Tibet animal populations.

Tibetan yaks basically live at an altitude of 5,000 m above sea level. High altitude is the most important ecological challenge for yaks. In this study, antibodies against *C. abortus* in yak varied from 8.0 to 18.2 % between regions. The highest seroprevalence for *C. abortus* was in Xiaqu township. The more intensive husbandry found in townships such as Xiaqu suggests that close contact at feeding may be a risk factor for the increased prevalence of *C. abortus*.

Among the different age groups, a higher prevalence (15.9 %) was detected in the 1 to 2 year age category but was not significant (*p* > 0.05), This pattern of age-related seroprevalence has also been observed in previous surveys of yaks elsewhere in China [16, 17] and was also not different from reports in cattle in Guangzhou, China [20]. High seroprevalence among members of the 1–2 years age category could be due to lower resistance or immunity, chronic infection, or sustained infection in yaks of this age.

Tibet is one of four major pastoral areas and an important grassland animal husbandry production base in China. There are about 4 million yaks in Tibet which makes it an economically important livestock species. Yaks abortion caused by *C. abortus* infection in Qinghai province has been reported previously [15]. However, the prevalence of *C. abortus* in yak population in the vast Tibet area is not known. The current study showed an overall serological positive ratio of 11.1 % by a *C. abortus* antibody-specific ELISA kits. According to local herdsmen, yak flocks suffered abortions in Tibet in recent years. We believe that some of the yak abortion cases might be the outcome of *C. abortus* infection in the yak population, although *C. abortus*-related abortion has not been investigated. *C. abortus* is a known zoonotic bacterial pathogen. Several reports have demonstrated that humans can acquire infection through contact with infected animals or aborted feteus and placenta, resulting in severe health problems including atypical pneumonia in men and abortion or pelvic inflammatory diseases in women [21, 22]. It might be a potential risk for public health.

In conclusion, the results showed a *C. abortus* seroprevalence of 11.1 % in yaks in Tibet, China. To our knowledge, the present report is the first to document the seroprevalence of *C. abortus* infection in yaks in Tibet, China. Further work is required to establish the significance of *C. abortus* infection in Tibetan yaks.

## Data Availability

Please contact the authors for data requests.

## Abbreviations

CI: Confidence interval
IHA: Indirect hemagglutination assay
ELISA: Enzyme-linked immunosorbent assay
